# Compliance with children’s television food advertising regulations in Australia

**DOI:** 10.1186/1471-2458-12-846

**Published:** 2012-10-05

**Authors:** Michele Roberts, Simone Pettigrew, Kathy Chapman, Caroline Miller, Pascale Quester

**Affiliations:** 1 The University of Western Australia (M263), 35 Stirling Highway, Crawley, WA, 6009, Australia; 2 Health Strategies, Cancer Council NSW, University of Sydney, Sydney, Australia; 3 South Australian Health and Medical Research Institute, University of Adelaide, Adelaide, SA, Australia; 4 Discipline of Public Health, School of Population Health and Clinical Practice, University of Adelaide, Adelaide, SA, Australia

**Keywords:** Child obesity, Food advertising, Regulatory compliance, Public policy, Diet, Nutrition, Food marketing

## Abstract

**Background:**

The objective of this study was to assess the effectiveness of the Australian co-regulatory system in limiting children’s exposure to unhealthy television food advertising by measuring compliance with mandatory and voluntary regulations. An audit was conducted on food and beverage television advertisements broadcast in five major Australian cities during children’s programming time from 1^st^ September 2010 to 31^st^ October 2010. The data were assessed against mandatory and voluntary advertising regulations, the information contained in an industry report of breaches, and the Australian Guide to Healthy Eating.

**Results:**

During the two months of data collection there were a total of 951 breaches of the combined regulations. This included 619 breaches of the mandatory regulations (CTS) and 332 breaches of the voluntary regulations (RCMI and QSRI). Almost 83% of all food and beverages advertised during children’s programming times were for foods classified as ‘Extras’ in the Australian Guide to Healthy Eating. There were also breaches in relation to the amount of advertising repetition and the use of promotional appeals such as premium offers, competitions, and endorsements by popular children’s characters. The self-regulatory systems were found to have flaws in their reporting and there were errors in the Australian Food and Grocery Council’s compliance report.

**Conclusions:**

This audit suggests that current advertising regulations are inadequate. Regulations need to be closely monitored and more tightly enforced to protect children from advertisements for unhealthy foods.

## Background

With a quarter of Australian children obese or overweight, the need to address all potential causes of obesity is paramount [1]. Evidence from international systematic reviews shows that food advertising can influence children’s food preferences and behaviours [2,3] and should therefore be addressed as part of any potential solution to childhood obesity.

The current system of regulating child-targeted television food advertising in Australia is comprised of both mandatory and self-regulatory elements. The mandatory regulations are embedded in the Children’s Television Standards (CTS) [4], which cover television viewing during C periods. These C periods are the times nominated by broadcasters as children’s viewing times. They must occur during C bands, which are time periods nominated by the regulator (the Australian Communications and Media Authority (ACMA)) as suitable for children’s viewing, and include 7.00-8.30 am and 4.00-8.30 pm on Monday to Friday and 7 am to 8.30 pm on Saturday, Sunday and school holidays [4]. The mandatory CTS were revised in 2010 to clarify the rules on premiums, reiterating the need for premiums to be incidental to the advertised product, and providing a definition of incidental [4]. The rules on popular characters and personalities in children’s advertising were also strengthened, in effect precluding these marketing devices from featuring in advertisements unless depicted on the product packaging [4].

In addition, two different sets of voluntary regulations were introduced by the food industry in 2009. The first was the Responsible Children's Marketing Initiative (RCMI), overseen by the Australian Food and Grocery Council (AFGC) [5]. The stated aim of this code of practice is to “Help promote healthy dietary choices and lifestyles to Australian children” (AFGC, 2009, web page). The signatories are 18 leading food manufacturers, including Coca Cola, Kraft, Nestlé, Mars, and Cadbury. The code covers television, radio, and the Internet, as well as licensed cartoon characters. Each food company must provide an individual action plan explaining how they will follow the core principles of the code and agree to advertise to children younger than 12 years only if the products are healthy and the advertising messages promote healthy lifestyles. The second set of voluntary regulations to be introduced was the Australian Quick Service Restaurant Industry’s (QSRI) Initiative for Responsible Advertising and Marketing to Children [6]. Signatories to this set of principles include most of the leading quick-service restaurant chains. The principles specify maximum levels of sugar, salt, and fat in advertised meals. The principles were developed in conjunction with the RCMI.

The current system of regulating child-targeted television food advertising in Australia has been criticised as lacking transparency because there is no published schedule of what constitutes children’s broadcasting times (C periods) [7,8]. Details of the times designated by broadcasters as children’s viewing times are contained in a schedule supplied annually by each broadcaster to the ACMA. These data are not readily available to the public, making it difficult to monitor the regulations, which are therefore prone to violations [7,9,10]. The advertising complaints system relies on complaints by the community to monitor adherence to the standards [11], an almost impossible task without knowing which timeslots are designated by each broadcaster as children’s viewing times.

No previous research appears to have analysed broadcast advertisements against the ACMA’s scheduling data, instead using times when children are likely to be viewing as a proxy for scheduling information. An example of this approach is a study that examined 64 hours of programs watched by substantial numbers of children and found that three-quarters of advertisements that included a premium breached the mandatory CTS requirement that any mention of the premium must be incidental to the main product [9]. Another study of 672 hours of programs watched by substantial numbers of children found breaches of the CTS on premium offers and misleading information in the form of suggestions that the advertised food could make children superior to their peers [10].

The present study involved a comprehensive review of advertisers’ compliance with Australia’s mandatory and self-regulatory systems to assess their effectiveness. The outcomes provide important information for policy makers in their efforts to evaluate the effectiveness of existing systems and their deliberations over future strategies to protect children from exposure to advertisements for unhealthy foods. The results also provide insights into the difficulties of monitoring and evaluating voluntary and compulsory advertising regulations.

## Methods

### Sample

Two months of television advertisements for food and beverages were purchased from a media monitoring company. The data covered 24 hours of television programming per day on the four main free to air channels in Australia’s five largest capital cities (Sydney, Melbourne, Brisbane, Perth, and Adelaide) from 1st September 2010 to 31st October 2010. The purchased data included all creative content, scheduling information, and media placement costs.

### Coding

A coding framework was developed that included items relating to aspects of the mandatory and voluntary regulations. The advertisements were analysed by two of the authors to identify the promotional techniques employed and to classify the promoted foods as healthy or unhealthy using the Australian Guide to Healthy Eating [12]. The authors coded the data independently and then compared all coding. Where disagreements were found in the two sets of coding, both authors re-examined the advertisements and reviewed the definitions from the CTS, which allowed consensus to be reached.

The data were analysed against the schedules for C periods provided by each broadcaster to the ACMA. These reports were obtained through the assistance of the ACMA, who had to contact each broadcaster to request permission prior to releasing the schedules. In addition, the data were assessed against voluntary industry codes, the information contained in industry reports of breaches, and the Australian Guide to Healthy Eating [12].

## Results

During the two months of data collection, 426 food and beverage advertisements were shown during C periods across the five cities (averaging 85 per city). These exposures related to 47 discrete advertising campaigns. There were a total of 951 breaches of the combined regulations identified in two months of children’s programming. This comprised 619 breaches of the mandatory regulations (CTS) and 332 breaches of the voluntary regulations (RCMI and QSRI). The high number of breaches relative to the number of exposures appears to have two main causes. In the first instance, there is substantial duplication of requirements between the mandatory and voluntary regulations. As a result, one exposure of one advertisement could be recorded as multiple breaches because it contravened multiple requirements within and between the codes. Second, there were high levels of repetition of advertisements featuring multiple breaches over the data collection period. Table 1 shows that over half the advertising that occurred during C periods originated from just two companies, Simplot and Golden Circle. Only two of the five heaviest advertisers during C periods, Simplot and McDonald’s, were signatories to either of the voluntary regulations.

**Table 1:** Top five heaviest advertisers during C periods

**Brand**	**Products**	**Number of aired advertisements**	**Percent**
Simplot Australia	Bird’s Eye frozen fish fingers	139	32.5
Golden Circle	LOL sweetened fruit juice	108	25.3
Subway	Filled rolls and sandwiches	39	9.1
Solar Eggs	Eggs	36	8.4
McDonald’s	Happy Meals (assorted food options)	25	5.9
Total		347	81.2%*

* Other companies accounted for 18.8% of advertisements (n = 79).

### Compliance with mandatory regulations

As noted above, a substantial number of breaches of the CTS were recorded (see Tables 2 and 3). Table 2 shows that there were six breaches of CTS 29 (“During any 30 minutes of a C period a licensee may broadcast the same advertisement no more than twice”). These breaches, however, were limited to two companies and one broadcaster.

**Table 2:** Breaches of Children’s Television Standard 29

**Brand/product**	**Campaign**	**Date**	**Time**	**Network**
CTS 29: - Repetition of advertisements: “During any 30 minutes of a C period a licensee may broadcast the same advertisement no more than twice”.				
Golden Circle	LOL/fruit juice, Make Me Famous Competition	11/09/10	11:07:18	Seven Network, Brisbane
			11:15:17	
			11:37:00	
Golden Circle	LOL/fruit juice, Make Me Famous Competition	18/09/10	11:06:56	Seven Network, Brisbane
			11:15:33	
			11:36:18	
Golden Circle	LOL/fruit juice, Make Me Famous Competition	20/09/10	02:54:58	Seven Network, Melbourne
			03:05:19	
			03:20:54	
Golden Circle	LOL/fruit juice, Make Me Famous Competition	24/09/10	04:08:15	Seven Network, Sydney
			04:15:26	
			04:24:48	
Simplot Australia	Bird’s Eye, animated pirate	27/09/10	02:40:10	Seven Network, Sydney
			02:46:40	
			03:04:24	
Simplot Australia	Bird’s Eye, animated pirate	28/09/10	02:40:42	Seven Network, Brisbane
			02:55:59	
			03:08:25	

**Table 3:** Breaches of Children’s Television Standards 33-35

**Brand/product**	**Campaign**	**Number of aired advertisements**
CTS 33(2a) - Premium offers: “Must not make reference to the premium in a way that is more than merely incidental to the reference to the advertised product or service” (n = 120).		
Golden Circle	LOL/fruit juice, Make Me Famous Competition	108
Ferrero Australia	Kinder Surprise confectionery	5
McDonald’s	Happy Meal with toys from Batman, ICarly and Australia Zoo	4
McDonald’s	What Song Makes You Feel Young?	2
Streets	Unlock a Free Magnum	1
CTS 33(2b) - Premium offers: “Must not stimulate any unreasonable expectation of the advertised product” (n = 3).		
McDonald’s	Happy Meal with Batman & ICarly toys	3
CTS 34(a) Competitions: “A summary of the basic rules must be stated” (n = 111).		
Golden Circle	LOL/fruit juice, Make Me Famous Competition	108
McDonald’s	What Song Makes You Feel Young?	2
Streets	Magnum ice cream stick, Unlock A Free Magnum	1
CTS 34(b): Competitions: “Any statement about the chance of winning must be clear, fair and accurate” (n = 110).		
Golden Circle	LOL/fruit juice, Make Me Famous Competition	108
McDonald's	What Song Makes You Feel Young?	2
CTS 35: Promotions and endorsements by popular characters: “No material broadcast during a C period may contain an endorsement, recommendation or promotion of a commercial product or service” (n = 269).		
Simplot	Bird’s Eye fish fingers with animated pirate	139
Golden Circle	LOL/fruit juice competition with Justice Crew	108
McDonald’s	Adult playground with Ronald McDonald and The Hamburgler	16
McDonald’s	Happy Meal toys from Batman, ICarly and Australia Zoo	4
Mamee	Monster noodle snack with animated monster	2
TOTAL		613

Table 3 shows that there were 613 breaches relating to premiums, competitions, and the use of popular characters. Six companies breached the relevant standards, although the heavy repetition of two campaigns resulted in the large number of exposures.

### Compliance with voluntary regulations

A critical mechanism for effective self-regulation is reporting on compliance levels. The AFGC has released the RCMI 2010 Compliance Report [13], but no report has been released to date for the QSRI. There were discrepancies between the results in the scheduling data obtained from broadcasters via the ACMA and the results published in the RCMI compliance report. The following companies reported that they had undertaken no promotion to children younger than 12 years of age, however advertisements were aired for their products during C periods: Ferrero (n = 8 repetitions), Coca Cola (n = 1), and Kraft (n = 1). Simplot reported ‘occasions’ where advertisements for fish fingers were inadvertently aired during C periods, but claimed to have taken steps to prevent further breaches. However, a total of 139 repetitions of this ad were found (see Table 3), indicating that the problem was more extensive than a few isolated instances.

Breaches of the voluntary regulations are documented in Tables 4 and 5. Compared to the RCMI, there were fewer breaches of the QSRI identified, partly because the latter has fewer signatories and partly because it is less stringent. The RCMI code requires the advertisement to represent a healthier choice *and* a healthy lifestyle, whereas the QSRI requires advertisements to represent *either* a healthier choice *or* a healthy lifestyle. In addition, most advertisements for McDonald’s shown during C periods were compliant because they were corporate advertisements that promoted the McDonald’s brand rather than specific foods.

**Table 4:** Breaches of the Responsible Children’s Marketing Initiative

**Regulation**	**Campaign breaches**	**Number of aired advertisements**
“Participants will not advertise food and beverage products to children under 12 in media unless:	Simplot; Bird’s Eye fish fingers	139
1. those products represent healthy dietary choices, consistent with established scientific or Australian government standards	Nestle: Milo cereal	5
	Ferrero: Kinder Surprise confectionery	5
**and**	Ferrero: Nutella chocolate spread	3
2. the advertising and/or marketing communication activities reference, or are in the context of, a healthy lifestyle, designed to appeal to the intended audience through messaging that encourages:	Fonterra: Mainland Butter Soft	2
	Fonterra: Mainland Cracker Cuts	1
·good dietary habits, consistent with established scientific or government criteria	Kraft: Oreo chocolate biscuits	1
·physical activity” (p 1–2).	Unilever: Street’s Magnum ice cream	1
Participants will not use popular personalities, program characters or licensed characters in advertising primarily directed to children under 12	Simplot Bird’s Eye: fish fingers animated pirate	139
Participants will commit to not advertising premium offers unless the reference to the premium is merely incidental to the product.	Ferrero Australia: Kinder Surprise	5
TOTAL		301

**Table 5:** Breaches of the Quick Service Restaurant Industry Initiative

**Relevant section of the Code**	**Campaign breaches**	**Number of aired advertisements**
“Advertising must:	Hungry Jack's: Angus Brekky Wrap	1
(a) Represent healthier choices, as determined by a defined set of Nutrition Criteria for assessing children’s meals [14]	McDonald’s: The Quarter Pounder	1
and/or	Red Rooster Free Range burger	1
	Red Rooster Free Range wrap	1
(b) Represent a healthy lifestyle, designed to appeal to the intended audience through messaging that encourages:	Red Rooster: Two For $10	1
(i) healthier choices, as determined by a defined set of Nutrition Criteria for assessing children’s meals and		
(ii) physical activity” (p 1–2).		
No premium offers unless the reference to the premium is merely incidental to the product.	McDonald’s Happy Meal with toys from Batman, ICarly and Australia Zoo	4
	McDonald's: What Song Makes You Feel Young?	2
No promotions and endorsements by popular characters	McDonald’s Adult playground with Ronald McDonald and Hamburger	16
	McDonald’s Happy Meal with toys from Batman, ICarly and Australia Zoo	4
TOTAL		29

### Discussion and conclusion

The data show that the current regulatory system in Australia is not providing comprehensive protection for children from exposure to television advertising for unhealthy foods. Breaches of the regulations are also leaving children exposed to promotional techniques such as premium offers and spokescharacters in advertisements for unhealthy foods.

Despite calls for more advertising regulations to protect children from unhealthy food advertising in Australia [14-16], it is clear that the current regulations are not effectively implemented and that they are breached and inadequately monitored. This indicates that much could be achieved by implementing tighter controls around existing regulations. However, this is difficult because the current regulatory systems are based on a schedule of children’s viewing times that is not available for public scrutiny. Although individual programs are identified within television viewing guides, this system is inadequate for generating the data required for large scale monitoring. The ACMA were not aware of ever having provided access to the schedule before, and written permission had to be obtained from each broadcaster before the information could be made available for this study. It is critical that any system of advertising regulation is transparent and open to public scrutiny. The findings of this analysis suggest that more effective advertising regulations could be achieved by simply reviewing the transparency and monitoring of current advertising regulations.

It should also be noted that the broadcaster’s scheduled C periods are not the times when most children are viewing. Television audience data from OzTam Australian Television Audience Measurement [14] indicate that larger numbers of children regularly view television outside these times. Therefore, these co-regulatory arrangements are not effective in eliminating children’s exposure to unhealthy food marketing during children’s popular viewing times [9,10,14].

These findings have importance beyond the regulatory situation in Australia. The USA and UK have both pursued a path of self-regulation of children’s advertising [17], and a review of regulatory systems in 20 developed countries found that voluntary codes and self-regulation were the usual approaches taken [18]. The present study illustrates the problems that can be associated with compliance and reporting, indicating the need to conduct similar studies elsewhere to better inform policy development. Although there have been several studies published on the extent and nature of advertising regulations throughout the world [19-22] and the impact of advertising bans [23], there is a vacuum of research on compliance with advertising regulations. Exceptions include a Spanish study on compliance with the self-regulatory advertising code that found high levels of non-compliance among both signatories and non-signatories [24], and an Australian study that found high levels of non-compliance with mandatory regulations governing the use of premiums in children’s advertising [9].

A limitation of this study is the use of a data collection period that fails to capture seasonal variations. However, compared to previous studies of regulation compliance that have utilised data sets ranging in duration from 63 hours [9] to 80 hours of programming [24], this was a relatively large sample that was purposively selected to cover both school holidays and term time. As such, it is likely that the data used in the study provide a representative snapshot of food and beverage advertising practices.

To conclude, it appears that more effective regulation of television food advertising would constitute an important element of the public policy response to child obesity. Self-regulation of food advertising by the food industry is falling short of its potential due to coverage of the voluntary codes being limited to signatory companies and inadequate compliance and reporting levels. In addition, there are numerous breaches of the mandatory Children’s Television Standards occurring, indicating that monitoring and enforcement of this form of mandatory regulation could also be more effective. It may thus be time to identify alternative means of protecting children from advertising for unhealthy foods.

## Competing interests

All authors declared that they have no competing interests.

## Authors’ contributions

All authors assisted in conceptualising the study and preparing the manuscript. MR and SP developed the coding framework and engaged in advertisement coding. MR took primary responsibility for the data analysis. All authors read and approved the final manuscript.

## Pre-publication history

The pre-publication history for this paper can be accessed here:

http://www.biomedcentral.com/1471-2458/12/846/prepub
